# Pain Self-Management with Inhaled Methoxyflurane by Emergency Department Trauma Patients: A Prospective, Interventional, Single-Center Study

**DOI:** 10.3390/ijerph20126107

**Published:** 2023-06-12

**Authors:** Daniel Aiham Ghazali, Donia Bouzid, Alix Frachon, Sarah Ait-Abdesselam, Philippe Kenway, Christophe Choquet, Enrique Casalino

**Affiliations:** 1 Emergency Department and Emergency Medical Services, Amiens University Medical Center, 1 Rond-Point du Professeur Christian Cabrol, 80000 Amiens, France; 2 DREAMS (Department of Research in Emergency Medicine and Simulation) Research Unit, Amiens University Medical Center and Jules Verne University of Picardie, 80000 Amiens, France; 3 IAME “Infection, Antimicrobials, Modelling, Evolution” Research Unit, INSERM UMR1137, University of Paris Cité, 75018 Paris, France; 4 Emergency Department, Bichat University Medical Center, 75018 Paris, France

**Keywords:** pain, trauma, numerical pain rating scale (NPRS), WHO, analgesic ladder, methoxyflurane

## Abstract

The coronavirus disease 2019 (COVID-19) pandemic has led to overcrowding in many emergency departments (EDs). The present single-center, prospective, interventional study (conducted at Bichat University Medical Center (Paris, France)) was designed to assess the impact of self-administered, inhaled, low-dose methoxyflurane on trauma pain in a pre-ED fast-track zone dedicated to the management of lower-acuity non-COVID-19 patients. In the first phase of the study, the control group consisted of patients with mild-to-moderate trauma pain, for whom the triage nurse initiated pain management (based on the World Health Organization (WHO)’s analgesic ladder). In the second phase, the intervention group consisted of similar patients who self-administered methoxyflurane as an adjuvant to the standard analgesic ladder. The primary endpoint was the numerical pain rating scale (NPRS) score (from 0 to 10) recorded at different time points during the patient’s care (T0: arrival in the ED, T1: exit from the triage box, T2: in the radiology department, T3: clinical examination, and T4: discharge from the ED). The level of agreement between the NPRS and the WHO analgesic ladder was assessed by the calculation of Cohen’s kappa. Pairwise comparisons of continuous variables were performed with Student’s *t*-test or a non-parametric Mann–Whitney U test. Changes over time in the NPRS were analyzed in an analysis of variance (with Scheffe’s post hoc test if a pairwise comparison was significant) or a non-parametric Kruskal–Wallis H test. In all, 268 and 252 patients were included in the control and intervention groups, respectively. The two groups had similar characteristics. The level of agreement between the NPRS score and the analgesic ladder was high in both the control and intervention groups (Cohen’s kappa: 0.74 and 0.70, respectively). The NPRS score decreased significantly between T0 and T4 in both groups (*p* < 0.001), but the decrease between T2 and T4 was significantly greater in the intervention group (*p* < 0.001). The proportion of patients still in pain on discharge was significantly lower in the intervention group than in the control group (*p* = 0.001). In conclusion, a combination of self-administered methoxyflurane and the WHO analgesic ladder improves pain management in the ED.

## 1. Introduction

Pain is the most common complaint by trauma patients in the ED [1,2]. Pain management can be influenced by a reduction in the time that ED staff devote to patients in general and lower-acuity patients in particular. Although optimal pain management is a key quality indicator, assessment of pain in EDs is irregular [3], and insufficient pain relief remains a problem [4]. Tcherny-Lessenot et al. report that pain is the reason for consultation in 80% of adult cases in the ED [5]. Guéant et al. have shown that the pain is severe in 40% of cases and that 30% of patients in severe pain did not receive any treatment [6]. Sufficient time, staff training, interdisciplinary cooperation, and patient involvement are key factors for efficient pain treatment [7]. Self-assessment of pain is essential but must be followed by the provision of analgesics [2]. In the ED, pain is assessed by the triage nurse as soon as the patient arrives; in principle, the nurse can start the treatment immediately. However, pain is underreported and is not optimally managed [7,8,9]. Methoxyflurane (Penthrox^®^, Medical Developments International Limited, Melbourne, Australia) is a volatile inhalation analgesic that provides rapid, short-term analgesia via a hand-held inhaler device [10] and may be an effective, non-narcotic option for emergency pain management in prehospital and ED settings [1].

When a patient arrives at an ED in France, he/she is ranked by a triage nurse from 1 (most severe) to 5 (least severe) according to the French Emergency Nurses Classification in Hospital scale (Classification Infirmière des Malades aux Urgences, CIMU) [11]. CIMU 1 patients are in an immediately life-threatening situation and require immediate medical intervention. CIMU 2 patients have a marked impairment of a vital organ or an imminently life-threatening or functionally disabling traumatic lesion; they require medical intervention within 20 min. CIMU 3 patients have a functional impairment or organic lesions likely to deteriorate within 24 h or are in a complex medical situation justifying the use of several hospital resources; these patients require medical intervention within 60 min. CIMU 4 patients are stable and have noncomplicated functional impairments or organ damage; however, their condition justifies the urgent use within 120 min (according to the French Emergency Medicine Society) of at least one hospital resource for diagnosis or treatment. CIMU 5 patients have no functional impairments or organ damage justifying the use of hospital resources for diagnosis or treatment. The French Emergency Medicine Society recommends that these patients should not have to wait for more than 240 min.

The COVID-19 pandemic prompted our institution (Bichat University Medical Center, Paris, France) to create a dedicated fast-track area outside the ED for the management of lower-acuity (CIMU 4 or 5), non-COVID-19 patients, in order to limit contact with COVID-19 patients. In the fast-track area, a triage nurse prescribed X-rays (if required and according to a predefined protocol) before the patient was examined by an emergency physician; the objective was to limit the length of stay in the ED. During the COVID-19 pandemic, interactions between patients and caregivers were restricted by the ED overcrowding. In this context, inhaled low-dose methoxyflurane [12,13] can be used for the self-administered emergency relief of moderate-to-severe trauma-related pain in adults [14]. This technique enables patients to manage their pain outside the ED (e.g., when they have to move for an X-ray examination). Methoxyflurane has a rapid onset of action: around 4 min, which corresponds to 6 to 10 inhalations [1,15]. The overall aim of the present study was to assess the impact of the use of methoxyflurane in the management of trauma pain in the ED. We, therefore, conducted a “before-after” study by comparing periods before and after the introduction of methoxyflurane in the triage nurses’ pain treatment protocol. The primary objective of the present study was to assess the impact of the use of inhaled low-dose methoxyflurane on trauma pain measured in the ED’s dedicated fast-track area for lower-acuity patients. The secondary objectives were to assess pain management in the ED (as set out in the department’s standard operating procedures for pain assessment and treatment guidance) and the patients’ levels of satisfaction with this pain management. Lastly, we assessed the triage nurses’ levels of satisfaction with the availability of methoxyflurane for pain management.

## 2. Methods

### 2.1. Study Design

This prospective, interventional (before/after) single-center study was conducted in the ED at Bichat University Medical Center from 1 January 2020, to 31 December 2021. The study complied with the principles of the Declaration of Helsinki and was registered at the Thai Clinical Trial Registry (a World Health Organization (WHO)-approved primary registry; trial reference number: TCTR20170910001). The protocol was approved by the investigational review board at Bichat University Medical Center and by the French National Agency for the Safety of Medicines and Health Products (ANSM; reference: 2017-A01073-50) as an evaluation of professional practice [16].

### 2.2. Population

On average, 250 patients per day attend the Bichat University Hospital’s ED (i.e., around 90,000 per year). One-third of the patients were managed on the fast track. The study included volunteer adult patients (aged 18 and over) admitted to the ED for painful, minor trauma (defined as trauma in the absence of life-threatening and/or severe injuries, such as fractures involving sites other than limbs, multiple or open fractures, vascular injury or nerve damage). Pain was rated on a numerical scale from 0 (no pain) to 10 (the worst imaginable pain). Only patients with mild pain (a numerical pain rating scale (NPRS) score ≤ 3) or moderate pain (an NPRS of 4 to 6) at baseline and who consented to participate in the study were included.

To ensure that the study groups were as homogeneous as possible, the following exclusion criteria were applied: (i) inability to self-assess pain (including the presence of neurological conditions, such as dementia, which made patients uncooperative); (ii) refusal of analgesic treatment; (iii) severe pain (an NPRS score ≥ 7) and their direct admission to the ED without an assessment by the triage nurse and without application of the pain relief protocol at the reception (thus avoiding selection bias); and (iv) analgesic treatment before arrival in the ED (e.g., provided by emergency medical services). In France, the emergency medical team includes an emergency physician and an emergency nurse, who can initiate pain management in prehospital settings.

### 2.3. Intervention

Pain was self-assessed (using the NPRS) at different time points during the patient’s management. The pain assessment document has been validated for this use (Appendix A) [16]. A nine-item questionnaire on satisfaction with pain management during the ED visit was also administered (Appendix B).

All the triage nurses had at least 6 months of experience and had been trained (using simulations) to (i) manage pain, (ii) explain the use of the pain self-questionnaire to the patient (in both study phases), and (iii) explain the use of the methoxyflurane inhalation device (in the second phase only) [8,16].

In the first phase of the study, the control group consisted of patients to whom the triage nurse provided pain management and who rated their pain level on the NPRS during their stay in the ED. Our analgesia protocol complied with the WHO’s three-step analgesic ladder and the ANSM’s guidelines [16]: (i) an NPRS score ≤ 3 (mild pain): treatment with a non-opioid analgesic (acetaminophen 1000 mg); (ii) an NPRS score of 4 to 6 (moderate pain): treatment with both acetaminophen (1000 mg) and a weak-opioid analgesic (codeine 60 mg); and (iii) an NPRS score ≥ 7 (severe pain): treatment with both acetaminophen and an opioid analgesic (morphine sulfate, 10 mg or (for patients over the age of 65 or those with kidney or liver failure) 5 mg).

In the second phase of the study (i.e., after the introduction of self-administered methoxyflurane treatment in our medical center), the intervention group consisted of patients who self-administered methoxyflurane as an adjuvant to the above-mentioned standard analgesic ladder.

For patients with an NPRS score < 3 while immobilized, methoxyflurane could be self-administered if the triage nurse felt that the patient’s pain level was likely to increase during manipulation for the X-ray (e.g., the presence of a dislocation or fracture). If a patient started to experience severe pain (i.e., NPRS score > 7, especially during positioning for X-rays), additional treatment with an opioid analgesic was initiated.

### 2.4. Provision of Information on Methoxyflurane and Use by the Patient

Methoxyflurane is an inhaled agent with analgesic, hypnotic, and mild anesthetic properties [12,13]. Although methoxyflurane was regularly used as a general anesthetic in the 1960s, its clinical role decreased gradually in the 1970s following reports of dose-dependent nephrotoxicity [17]. Low doses (3 to 6 mL) of methoxyflurane have been used for analgesia in patients with post-traumatic pain for several decades in Australia and New Zealand, and this treatment was recently introduced in Europe. In France, methoxyflurane received marketing approval in November 2016, with an indication of moderate-to-severe trauma-related pain in conscious adult patients [18]. Methoxyflurane is highly lipophilic and has an extremely rapid analgesic onset of action [15]. The compound is metabolized in the liver. The main adverse events (AEs) associated with the use of methoxyflurane are hepatotoxicity and dose-dependent nephrotoxicity. However, these AEs were not reported during the use of over 5 million doses of inhaled methoxyflurane [10].

In the present study, one or two 3 mL vials of methoxyflurane could be used by the patient. The patient’s pain level was systematically reassessed after administration of the first vial. The nurse prepared the methoxyflurane inhaler and showed the patient how to use it. The patient inhaled and exhaled through the inhaler several times upon arrival in the ED. The triage nurse instructed the patient to inhale methoxyflurane before each mobilization (e.g., for an X-ray) but specified that he/she did not have to inhale the analgesic all the time. Our instructions on the use of methoxyflurane were based on the guidelines issued by the French National Agency for the Safety of Medicines and Health Products (ANSM) [19].

### 2.5. Data Collection, Outcomes, and Comparisons

The triage nurse handed out the pain assessment document in French or English (Appendix A). The pain assessment document comprised three sections [8,16]: two sections were filled out by the triage nurse, and one part was filled out by the patient during their stay in the ED. The document was used to record information on the nature of the trauma, the type of analgesics received, the time required for the onset of analgesia, the number of vials of methoxyflurane used (1 or 2), and any nonserious and serious AEs. The NPRS score was noted at five time points during the patient’s stay in the ED: arrival (T0), exit from the triage area (T1), imaging (T2), clinical examination (T3), and discharge (T4).

Lastly, the pain assessment document included a six-item satisfaction questionnaire. Each question was rated on a four-point Likert scale (very dissatisfied, dissatisfied, satisfied, or very satisfied). An overall satisfaction score (on a scale of 0 (not at all satisfied) to 10 (totally satisfied)) was also calculated. The completed survey was handed back to the fast-track physician at the end of the stay in the ED. Additional data were extracted from the hospital’s electronic health records using UrQual^®^ software version 4.0 (Maincare Solutions, Cestas, France). These data included sociodemographic variables, the date of ED attendance, the length of stay in the ED, and the reasons for ED attendance. The triage nurses’ level of satisfaction (rated from 0 (low) to 10 (high) with the availability of methoxyflurane for pain management was surveyed via an online questionnaire (Appendix C)). The control and intervention groups were compared with regard to all the variables collected.

### 2.6. Statistics

Anonymized data were collected using Excel 2016 (Microsoft^®^ Corporation, Redmond, WA, USA). Statistical analysis was performed using Statview^®^ software (version 4.5, SAS Institute Inc., Cary, NC, USA). The sample size (234 patients in each of the two groups) was calculated so that we could demonstrate an intergroup difference in the final pain level. The calculation was based on the results of a preliminary study in which adjuvant treatment with methoxyflurane for moderate-to-severe pain was associated with a lower mean NPRS score (4 out of 10 vs. 5 out of 10; standard deviation (SD): 3), with an alpha risk of 0.05, a statistical power of 0.95, and the use of two-tailed tests. The Kolmogorov–Smirnov test was used to determine whether or not the data on the study variables were normally distributed. Continuous variables were described as the mean ± SD or the median (interquartile range (IQR)), and categorical variables were described as the number (percentage). Continuous variables were compared in a series of pairwise comparisons, using Student’s *t*-test or a non-parametric Mann–Whitney U test if necessary. The changes over time in the NPRS were analyzed with the non-parametric Kruskal–Wallis H-test because the NRPS score was categorized as an ordinal variable. Categorical variables were compared in a chi-squared test. Cohen’s kappa was calculated as a guide to the level of agreement between the NPRS score and the analgesic ladder. The threshold for statistical significance was set to *p* < 0.05.

## 3. Results

### 3.1. Population

**Figure 1:**
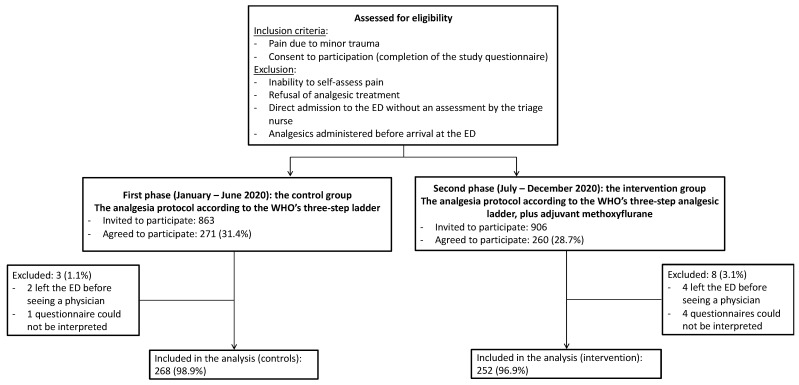
Study flow chart.

In all, 271 and 260 patients were included in the control and intervention groups, respectively. In the control group, two patients left the ED before the consultation, and one questionnaire was not usable. In the intervention group, four patients left the ED before the consultation, and four questionnaires were not usable. Hence, our final analysis included 268 patients in the control group and 252 in the intervention group (Figure 1).

The median (IQR) age (in years) was 31 [24; 44] in the control group and 32 [25; 42] in the intervention group (*p* = 0.95). The sex ratios were similar, with 110 (41%) women in the control group and 88 (35%) women in the intervention group (*p* = 0.14). The types of trauma were very similar in the two groups (*p* = 0.75) (Table 1).

The intervention group and control groups did not differ significantly in the time taken to initiate analgesic treatment, i.e., the time interval between the patient’s arrival in the triage box and the initiation of analgesic treatment. The median (IQR) time (in minutes) was 5 [0; 10] in the intervention group and 5 [0; 8] in the control group (*p* = 0.17). The length of stay in the ED was similar in the control and intervention groups: 114 [102; 129] and 110 [102; 127] minutes, respectively (*p* = 0.15). Only four of the patients in the intervention group used a second vial of methoxyflurane.

### 3.2. Analgesic Treatments Received

In the control group, 101 (37.7%) patients received a non-opioid analgesic, and 126 (47.0%) received a weak opioid. In the intervention group, 110 (43.7%) patients received (in addition to inhaled methoxyflurane) a non-opioid analgesic, and 121 (48.0%) received a weak opioid. The differences in these proportions were not statistically significant (Table 2). The proportion of patients having subsequently received additional opioid treatment in the ED was significantly higher in the control group than in the intervention group: n = 41 (15.3%) and 21 (8.3%), respectively (*p* = 0.01) (Table 2).

### 3.3. Adverse Events and Serious Adverse Events Related to Methoxyflurane

In the intervention group, the most frequent AEs related to methoxyflurane were somnolence (in 6% of patients), nausea (in 12%), and dizziness (in 3%). All these AEs resolved upon discontinuation of methoxyflurane administration.

### 3.4. Pain Self-Assessment during the Stay in the ED

On arrival in the ED, the NPRS score was 8 [7; 9] in the control group and 8 [6; 9] and the intervention group (*p* = 0.13). The minimum NRPS score was three in the control group and two in the intervention group. At discharge from the ED, the NRPS score had decreased by 5 points in the control group and by 6 points in the intervention group: the score was 3 [2; 4] in the control group and 2 [1; 2] in the intervention group (*p* < 0.0001).

**Figure 2:**
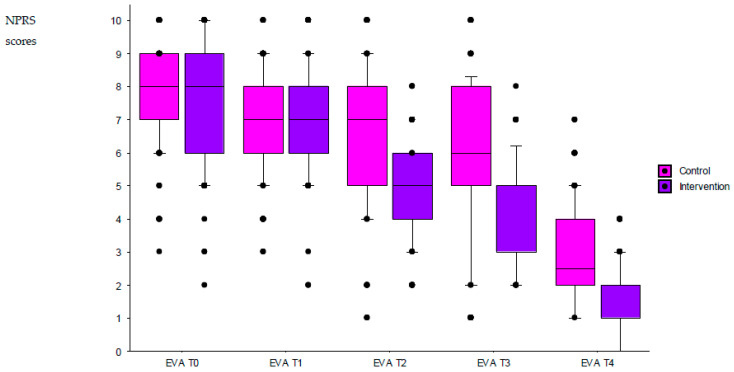
Changes in the NPRS scores in the two groups during patient management in the ED. Legend: EVA: evaluation time point; NPRS: numerical pain rating scale; T0: arrival in the ED; T1: exit from the triage area; T2: during imaging; T3: during a clinical examination; and T4: discharge from the ED (T4). The box plot displays the minimum, 10th percentile, 25th percentile (first quartile), median, 75th percentile (third quartile), and maximum.

An intragroup analysis over time showed that the NPRS scores decreased significantly between T0 and T4 in both the control group (H = 420.0; *p* < 0.001) and the intervention group (H = 601.9; *p* < 0.001) groups (Figure 2).

An intergroup comparison showed that the NPRS score decreased more in the intervention group than in the control group (H = 977.7; *p* < 0.001). Pairwise comparisons of the intervention vs. control groups at each time point showed that the NPRS scores were similar at T0 (i.e., on arrival at the ED; *p* = 0.13) and T1 (i.e., upon exit from the triage area; *p* = 0.61). In contrast, there was a significant difference at T2, T3, and T4 (i.e., during imaging, during the clinical examination, and on discharge from the ED; *p* < 0.0001 for all).

Of the 252 patients in the intervention group who completed the NPRS at discharge from the ED, only six (2.4%) had a score ≥4. In the control group, 21 out of 268 (7.8%) patients left the ED with an NPRS score ≥4; the difference between the intervention group and the control group was statistically significant (*p* = 0.005).

### 3.5. Quality of Pain Assessment and Pain Management

We assessed compliance with the ED’s analgesic protocol, i.e., the agreement between the NPRS score and the type of analgesic given: level 1 analgesics should be given when the NPRS score is ≤3, level 2 analgesics should be given when the NPRS score is between 3 and 6, and level 3 analgesics should be given when the NPRS score is ≥7. The percentage agreement between the NPRS score and the analgesic ladder was 86.6% in the control group (Cohen’s kappa: 0.74) and 84.9% in the intervention group (Cohen’s kappa: 0.70).

When considering the patients with minor trauma, 39 (14.6%) in the control group and 31 (12.3%) in the intervention group received more powerful analgesic treatment in the examination room (i.e., other than the treatment provided by the triage nurse); this intergroup difference was not significant (*p* = 0.36).

### 3.6. Assessment of Patient Satisfaction

The intergroup difference in the patients’ mean level of satisfaction with pain assessment by the triage nurse was not statistically significant (Table 3). However, patients were more satisfied with the analgesic treatment initiated by the triage nurse in the intervention group in comparison with the control group (*p* = 0.008). Statistically significant intergroup differences were noted for other aspects of pain management: overall satisfaction and the effectiveness of analgesics—especially during the imaging procedures were higher in the intervention group in comparison with the control group (*p* < 0.0001 for all the questions).

### 3.7. The Triage Nurses’ Levels of Satisfaction 

The triage nurses felt that patients in the ED fast track should have an opportunity to use adjuvant pain treatment (mean ± SD score: 8.6 ± 1.2). They considered that it was useful to have a self-administered treatment for pain management in a fast track where patients had less contact with the ED team (8.4 ± 1.2). The triage nurses were satisfied with the availability of methoxyflurane (9.1 ± 1.1) for relieving pain, in addition to the standard of care. They felt that after training, it was easy to explain to the patients how methoxyflurane should be used (8.2 ± 1.2). Lastly, they felt that the overall quality of pain management in the ED was improved by the convenience of having methoxyflurane for pain management (8.8 ± 1.1).

## 4. Discussion

The present study evaluated the value of adding self-administered treatments to the usual pain relief protocol applied by triage nurses upon the patient’s arrival in the ED. It is known that the assessment and treatment of pain in the ED are still inadequate [1,20]. Overcrowding in the ED makes it difficult to re-evaluate patients and, therefore, institute additional analgesic treatments. During the COVID-19 pandemic, the creation of a fast track for lower-acuity, non-COVID-19 patients outside the ED [21] prompted us to empower these individuals in their pain assessment [8]. Furthermore, we introduced methoxyflurane self-administration as an adjunct to standard analgesic treatments for mild and moderate trauma pain [1,15]. Methoxyflurane’s efficacy has been proven [22], and its use in a specific care pathway has been recommended for the management of acute traumatic pain [23]. As an adjuvant to the WHO analgesic ladder, methoxyflurane provides safe, short-term pain relief (for up to 30 min) [24].

When a patient can self-manage his/her adjunct analgesia, the ED’s physicians and nurses have more time to devote to other tasks [23]. In our study, the control and intervention groups were similar with regard to their demographic characteristics, the pain levels on entry and exit from the triage zone, the level of compliance with the ED’s protocol, the analgesics prescribed within the same time frame, and the same quality of care. In view of the similarities between the two groups and our application of a number of exclusion criteria, the differences observed upon exit from the triage area can reasonably be attributed to the use of methoxyflurane as an adjuvant treatment. The changes over time in pain scores showed that adjuvant analgesia with methoxyflurane and properly prescribed standard analgesia meant that most of the patients in the intervention group left the ED with an NPRS ≤ 3. In the control group, nearly one-third of patients had an NPRS greater than 4 at the same time point. Thus, methoxyflurane was associated with a reduction in pain levels by one-third when comparing the two groups [25]. Coffey et al. also found this degree of effectiveness, with a pain reduction of more than 30% [15]. Similarly, Fabbri et al. reported lower pain levels at a time point after the triage box [26]; in a study of 305 patients, the researchers demonstrated that the decrease in pain was significantly greater in the methoxyflurane group at 5, 10, 15 and 20 min and that these decreases were independent of the baseline pain score and the analgesic class administered [26]. In contrast, other studies have reached more mixed conclusions. In a meta-analysis of effectiveness data, Hong Liu et al. could not conclude with sufficient certainty that inhaled methoxyflurane was superior to standard analgesic treatment [27].

Most patients did not use a second vial of methoxyflurane; this absence might have helped to limit the occurrence of AEs. There are two possible explanations for the predominant use of a single vial: the relatively short duration of treatment and the use of multimodal analgesia. Firstly, the reorganization of our ED during the COVID-19 pandemic was associated with a reduction in the time to treatment and the duration of treatment for non-COVID-19 patients in the fast-track area [21]. The fast-track protocols allowed the triage nurse to start treating the pain as soon as the patients were admitted. Secondly, direct referral to the radiology department for certain limb X-rays before the clinical examination reduced the number of steps in the patient’s care pathway and thus reduced the length of stay in the ED. Mercadante et al. found that in patients requesting a second vial, the median (range) time between the administration of the first inhaler and that of the second inhaler was 54 (30–120) min) [1]. The AEs observed in the present study should prompt the introduction of safety rules to avoid aspiration—even when only a single vial is used.

Satisfaction with the analgesia received from the triage nurse and with the effectiveness of the analgesia (particularly during imaging) and overall satisfaction were significantly greater in the intervention group. This finding was in line with the data from the literature on greater satisfaction when methoxyflurane was used [25]. Our present results stand out because they highlight the value of methoxyflurane self-administration at times when the ED staff have less access to the patient complex (e.g., during imaging examinations outside the ED). Moreover, the results of the present study highlighted the importance of having a protocol for managing pain immediately upon arrival in the ED. When multimodal pain management was initiated by the triage nurse, a few of the trauma patients required additional (second-line) analgesic treatment in the ED. Another hypothesis is that overcrowding leads to the underuse of second-line analgesic treatment. However, the low NRPS scores noted during the clinical examination and on discharge from ED in the interventional group suggest that the absence of additional analgesic treatment in the ED was due to the effectiveness of the treatment initiated by the triage nurse.

Lastly, our evaluation of the triage nurses’ professional practice revealed that methoxyflurane was easy to use. In the context of the COVID-19 pandemic, the triage nurses were pleased to be able to empower the patient via the self-management of pain. The objectives of this approach were to keep patients out of the ED as much as possible and to reduce the length of stay in the ED. Moreover, given the context of overcrowding in the ED, methoxyflurane allowed the pain to be managed in the absence of extensive contact with the ED’s physicians and nurses. Although the implementation of this procedure was prompted by the COVID-19 pandemic, methoxyflurane self-treatment can nevertheless be considered outside a pandemic context. The introduction of nurse-initiated analgesic care protocols helps to improve pain management and might accelerate patient flow in the ED.

### Study Limitations

The initial phase of the present study (the training of triage nurses) took place in 2019 in two EDs. However, due to the COVID-19 pandemic, methoxyflurane was introduced in one of the two only. Therefore, the pain management protocol at this site was analyzed by comparing the periods before vs. after the inclusion of methoxyflurane in the treatments that the triage nurse could deliver at the reception. The before/after study design was limited by the fact that all the patients were not managed by the same teams of triage nurses. This limitation is mitigated by the fact that all the triage nurses (regardless of the phase of the study) were trained in pain management and in patient empowerment for self-assessment. This training allowed us to standardize pain management in the ED and improve patient satisfaction [8]. The present study was subject to selection bias because patient inclusion was voluntary and systematic. It is possible that during periods of overcrowding, the triage nurses were less likely to invite patients to participate in the study. Furthermore, we recorded AEs associated with methoxyflurane but not those associated with other analgesics (especially opioids); this prevented us from comparing the intervention and control groups with regard to AEs. Lastly, the study’s single-center design might limit the external validity of our results because pain management practices differ from one ED to another ED. 

## 5. Conclusions

Overcrowding in EDs can lead to nonoptimal pain assessment and, thus, oligoanalgesia. Patient empowerment and self-administered methoxyflurane analgesia improve pain management and increase patient satisfaction. Standardized, patient-managed, multimodal analgesia initiated by trained triage nurses at the beginning of the care pathway represents a major advance in pain management for ED patients.

## Figures and Tables

**Table 1:** Trauma categories in the two study groups.

Trauma Category	Control Group (n, %)	Intervention Group (n, %)	*p*-Value
Contusion	43 (16.0)	31 (12.3)	0.22
Sprain	42 (15.7)	35 (13.9)	0.57
Fracture	106 (39.6)	88 (34.9)	0.27
Dislocation	54 (20.1)	69 (27.4)	0.06
Wounds	23 (8.6)	29 (11.5)	0.26
Total	268 (100)	252 (100)	

**Table 2:** Analgesic treatment in the two study groups.

Analgesic	Control Group (n, %)	Intervention Group (n, %)	*p*-Value
**Non-opioid analgesics**	101 (37.7)	110 (43.7)	0.17
**Weak opioids**	126 (47.0)	121 (48.0)	0.82
**Non-weak opioids**	41 15.3)	21 (8.3)	0.01
**Total**	268 (100)	252 (100)	

**Table 3:** The quality of pain management in the ED, according to a self-questionnaire completed by the patients.

Questionnaire Topic (Score Out of 10)	Control Group (Mean ± SD)	Intervention Group (Mean ± SD)	*p*
Quality of the pain assessment by the triage nurse on arrival in the triage area	7.7 ± 1.1	7.5 ± 1.2	0.26
Analgesic treatment initiated by the triage nurse in the triage area	6.8 ± 1.6	7.6 ± 1.1	0.008
Satisfaction with the available pain treatment	6.7 ± 1.3	8.1 ± 1.0	<0.0001
Effectiveness of the treatment when being positioned for imaging procedures	6.2 ± 1.4	8.3 ± 1.1	<0.0001
Overall effectiveness of the analgesic treatment	7.0 ± 1.3	8.4 ± 1.1	<0.0001
Overall satisfaction with pain management in the ED	6.7 ± 1.1	8.2 ± 1.2	<0.0001

**Figure A1:**
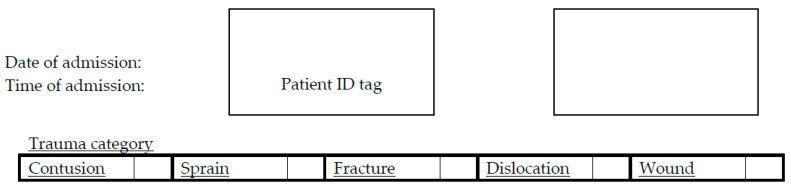
Pain assessment document.

## Data Availability

The materials described in the manuscript, including all relevant raw data, are freely available to any scientist wishing to use them for non-commercial purposes, without breaching participant confidentiality. For more details, please contact the corresponding author.

## Appendix A. Document for Pain Self-Assessment in the Emergency Department

Pain scale (rated by the patient) from 0 (no pain) to 10 (the worst imaginable pain).

Please circle the number that corresponds to your pain level.

Pain relief (completed by the ED staff): Acetaminophen (dose ___) Acetaminophen—codeine (dose ___) Morphine (dose ___). Time when analgesic was administered: ………………

Penthrox*

Number of inhalers/vials administered: 1 2

Duration of Penthrox* use by the patient: <15 min 15–30 min 30–90 min

## Appendix B. Questionnaire on the Patient’s Satisfaction with Pain Management during the ED Visit

Dear Sir or Madam,

You are attending the emergency department at Bichat University Hospital. Please take a few minutes to answer this survey. This will enable us to improve our procedures and the quality of care. Please rate each item from 0 (totally unsatisfied) to 10 (totally satisfied). Thank you for your help.

**Table A1:** Self-assessment of satisfaction.

Questions	Satisfaction (0–10)
Quality of the pain assessment by the triage nurse on arrival in the triage area	
Analgesic treatment initiated by the triage nurse in the triage area	
Satisfaction with the available pain treatment	
Effectiveness of the treatment when being positioned for imaging procedures	
Overall effectiveness of the analgesic treatment	
Overall satisfaction with pain management in the ED	

## Appendix C. Online Questionnaire for Triage Nurses

Please complete this questionnaire about your pain management practice in the fast-track for non-COVID-19 patients. Each statement must be rated from 0 (you totally disagree) to 10 (you totally agree).

1. Non-COVID-19 patients in the fast-track area must have an opportunity to use adjuvant pain treatment.
2. It is useful to have the option of self-administered pain management in the fast track.
3. I am satisfied with the option of having methoxyflurane for self-administered pain management in addition to the standard of care.
4. After training, it is easy to explain to patients how methoxyflurane should be used.
5. The overall quality of pain management in the ED was improved by the convenience of having methoxyflurane as a treatment option.
